# A new Bihari inequality and initial value problems of first order fractional differential equations

**DOI:** 10.1007/s13540-023-00152-5

**Published:** 2023-04-17

**Authors:** Kunquan Lan, J. R. L. Webb

**Affiliations:** 1 Department of Mathematics, Toronto Metropolitan University, Toronto, ON M5B 2K3 Canada; 2 School of Mathematics and Statistics, University of Glasgow, Glasgow, G12 8SQ UK

**Keywords:** Fractional equations, Initial value problems, Bihari inequality, global existence, 34A08, 26D10 (primary)and 34B18, 47H11, 47H30 (secondary )

## Abstract

We prove existence of solutions, and particularly positive solutions, of initial value problems (IVPs) for nonlinear fractional differential equations involving the Caputo differential operator of order $\alpha \in (0,1)$. One novelty in this paper is that it is not assumed that *f* is continuous but that it satisfies an $L^{p}$-Carathéodory condition for some $p>\frac{1}{\alpha }$ (detailed definitions are given in the paper). We prove existence on an interval [0, *T*] in cases where *T* can be arbitrarily large, called global solutions. The necessary *a priori* bounds are found using a new version of the Bihari inequality that we prove here. We show that global solutions exist when *f*(*t*, *u*) grows at most linearly in *u*, and also in some cases when the growth is faster than linear. We give examples of the new results for some fractional differential equations with nonlinearities related to some that occur in combustion theory. We also discuss in detail the often used alternative definition of Caputo fractional derivative and we show that it has severe disadvantages which restricts its use. In particular we prove that there is a necessary condition in order that solutions of the IVP can exist with this definition, which has often been overlooked in the literature.

## Introduction

We study the existence of continuous solutions, and particularly non-negative solutions, of initial value problems (IVPs) for nonlinear fractional differential equations (FDEs) involving the Caputo fractional differential operator of order $\alpha \in (0,1)$, denoted $D^{\alpha }_{*}$, as in [6, Definition 3.2] (precise definitions are given in Sect. 2), which is called a first order FDE in [18–20].

One of our goals is to prove existence for all *t* in an interval [0, *T*], especially in cases where *T* can be arbitrarily large, which we call global solutions.

We will employ the standard method of studying solutions via fixed points of the associated Volterra integral operator *N* acting in the space of continuous functions, using fixed point index theory or degree theory.

The IVP is written $D^{\alpha }_{*}u(t)=f(t,u(t))$ for almost every (a.e.) $t \in [0,T]$, $u(0)=u_{0}$, where $D^{\alpha }_{*}u$ is the Caputo differential operator of a continuous function *u* which satisfies an extra condition so that $D^{\alpha }_{*}u(t)$ exists a.e., and *N* is a Volterra integral operator $Nu(t)=u_{0}+\frac{1}{\Gamma (\alpha )}\int _0^t (t-s)^{\alpha -1}f(s,u(s))\,ds$ involving an integral with a singular kernel. Most previous works assume that *f* is continuous. We suppose that *f* satisfies an $L^{p}$-Carathéodory condition for some $p>\frac{1}{\alpha }$ (definition is given in Sect. 2), a weaker condition than continuity. We will rigorously prove the equivalence of solutions of the IVP and fixed points of *N* in our case.

The IVP was studied in Diethelm and Ford [7], existence and uniqueness results are proved in that paper when *f* satisfies a Lipschitz condition in the *u* variable. In the book of Diethelm [6], a local existence theorem (that is, a solution exists on some (possibly short) interval [0, *h*]) is proved when *f* is continuous, see [6, Theorem 6.1]. A global existence result is proved in [6, Corollary 6.3], when the nonlinearity *f* satisfies a sublinear growth assumption

1.1 $$\begin{aligned} |f(t,u)|\le c_{1}+c_{2} |u|^{\mu }\;\text {for each}\; t \in [0,T] \;\text {and} \; u\in \mathbb {R}, \end{aligned}$$

where $\mu \in (0,1)$, $c_{1}, c_{2}\in \mathbb {R}_{+}$ and $f:[0,T]\times \mathbb {R}\rightarrow \mathbb {R}$ is continuous.

The existence of global non-negative solutions for the IVP was studied in [29, Theorem 4.8] under the following weaker conditions on *f*,

1.2 $$\begin{aligned} f(t,u)=t^{-\gamma }g(t,u)\le M t^{-\gamma }(1+u)\; \text {for} \;(t,u)\in [0,T]\times \mathbb {R}_{+}, \end{aligned}$$

where $M>0$, $\gamma \in [0,\alpha )$ and $g:[0,T]\times \mathbb {R}_{+}\rightarrow \mathbb {R}_{+}$ is continuous, so *f* has a singularity when $t=0$. A new Gronwall inequality, suitable for fractional problems, and involving the exponential function, was proved in [29] and used to obtain a priori bounds.

Eloe and Masthay [10] consider an IVP for a problem with $\alpha \in (n-1,n]$ $n \in \mathbb {N}$, with a nonlinearity which depends on classical derivatives of order at most $n-1$. They establish a Peano type local existence theorem, a Picard type existence and uniqueness theorem, and give some results related to maximal intervals of smooth solutions.

Lakshmikantham and Vatsala [17] investigate the IVP with *f* continuous and discuss maximal and minimal solutions assuming a Hölder condition is satisfied, and they give a global existence result assuming that a maximal solution exists globally.

One of our main aims is to treat more general nonlinearities, $f:[0,T]\times \mathbb {R} \rightarrow \mathbb {R}$ is assumed to be $L^{p}$-Carathéodory as in Definition 2 below. We also have less restrictive conditions on the growth of *f* in the *u* variable than (1.1) and (1.2) by using a new Bihari inequality, replacing a continuity hypothesis by an integrability one, that we prove here. We will prove a general existence theorem which shows that global solutions exist when *f* grows at most linearly in *u*, and we have local existence when there is superlinear growth of the form $u^{r}$ for $r>1$. A new result we obtain is that global solutions can also exist in some cases when the growth is faster than linear.

The pioneering work on obtaining a priori bounds for fractional problems was done by Henry [14], this is applicable to functions with linear growth, the obtained bounds involve a Mittag-Leffler function. Many generalizations have been given, but we are not aware of any bounds involving Mittag-Leffler functions which are useful in our case. Medved$^\prime$ [22] gave several Bihari type inequalities in the continuous case, with a more complicated appearance than our result. Zhu [34, Theorem 3.1] proved some results similar to ours under continuity conditions. Zhu [33] has also given inequalities of Gronwall type involving the exponential function in the linear growth case.

As an example of our results, we obtain results on the existence of non-negative solutions of the IVP for first order FDEs with nonlinearities of a type that occur in combustion theory.

Many papers in the literature have studied solutions of Caputo FDEs with an often used definition of Caputo fractional derivative, denoted in this paper $D^{\alpha }_{C}$, which was given in Caputo [3, Eq.(5)]. The mathematical discussions of IVPs usually seek solutions in *C*[0, *T*] of the corresponding integral equation, where *f*(*t*, *u*) is only assumed to be continuous. We discuss the definition $D^{\alpha }_{C}$ in detail in Sect. 9 and we show that it has severe disadvantages. In particular we show that there is a necessary condition in order that solutions of the IVP can exist with this definition, which fact has often been overlooked in the literature. There are many published papers that claim an equivalence between the IVP and the integral equation, but this is false with the definition $D^{\alpha }_{C}$ without the necessary condition, and there are no known conditions on a function *f*(*t*, *u*) in order for the necessary condition to be satisfied by *Fu* (where $Fu(t)=f(t,u(t))$, the Nemytskii operator). The related results in these paper therefore lack a sound foundation, the definition $D^{\alpha }_{C}u$ for $\alpha \in (0,1)$ should not be used in the nonlinear case.

Fortunately, the definition of Caputo differential operator $D^{\alpha }_{*}u$, as in the well-known texts Diethelm [6, Definition 3.2], Kilbas, Srivastava and Trujillo (KST) [15, p. 91], is appropriate, and this is the definition we shall use to prove our results.

## Preliminaries

In order to have simpler formulas we consider functions defined on an arbitrary finite interval [0, *T*], which is equivalent to any finite interval [*a*, *b*] by the change of variable (simple translation) $\tau =a+t$ for $t \in [0,T]$ where $T=b-a$.

In this paper it is implicit that all functions are measurable, and that all integrals are Lebesgue integrals; almost everywhere (and almost every) will be abbreviated a.e.. For $1\le p<\infty$, $L^{p}=L^{p}[0,T]$ denotes the usual space of functions whose *p*-th power is Lebesgue integrable endowed with the norm $\Vert f\Vert _{p}:=\bigl (\int _0^T |f(s)|^{p}\,ds\bigr )^{1/p}$ and $L^{\infty }$ is the space of essentially bounded functions with the norm $\Vert f\Vert _{\infty }:={\text {esssup}}_{t \in [0,T]}|f(t)|$.

The space of functions that are continuous on [0, *T*] is denoted by *C*[0, *T*] or sometimes simply *C* and is endowed with the supremum norm $\Vert u\Vert _{\infty }:=\max _{t \in [0,T]}|u(t)|$. $C^1=C^{1}[0,T]$ denotes the space of continuously differentiable functions, first order derivatives exist and are continuous. A subscript $+$ will denote those functions in the corresponding space that are non-negative (a.e. in $L^{p}$ spaces). For $0<\alpha <1$, the Hölder space $C^{0,\alpha }[0,T]$, consists of all functions *f* such that $|f(t)-f(\tau )|\le M |t-\tau |^{\alpha }$ for some constant $M>0$.

We will also use the space of absolutely continuous functions which is denoted $AC=AC[0,T]$. The space *AC* is the appropriate space for the fundamental theorem of the calculus for Lebesgue integrals. In fact, we have the following equivalence.

2.1 $$\begin{aligned} \begin{aligned} u \in AC[0,T] \quad \text {if and only if u'(t) exists for a.e.} \;t \in [0,T]\\ \text {with} \; u' \in L^1[0,T]\text { and } u(t)-u(0)=\int _0^t u'(s)\,ds \;\;\text {for all} \; t \in [0,T]. \end{aligned} \end{aligned}$$

We write $g \in \textrm{Lip}$ and say that *g* is Lipschitz (or satisfies a Lipschitz condition) on its domain ${\text {dom}}(g)$ if there is a constant $L>0$ such that $|g(u)-g(v)| \le L|u-v|$ for all $u,v \in {\text {dom}}(g)$.

The following strict inclusions are well known (on a closed bounded interval).

$$\begin{aligned} C^{1} \subset \textrm{Lip} \subset AC \subset \text {differentiable a.e.},\\ AC \subset \text {uniformly continuous} \subset C. \end{aligned}$$

It is also known that, on a bounded interval, the sum and pointwise product of functions in *AC* belong to *AC* and if $u \in AC$ and $g \in \textrm{Lip}$ then the composition $g \circ u \in AC$, but the composition of *AC* functions need not be *AC*.

Note that there are functions that are Hölder continuous but are not *AC*, for example a Weierstrass function, and *AC* functions that are not Hölder continuous.

### Definition 1

A function $f:[0,T] \times \mathbb {R} \rightarrow \mathbb {R}$ (or $f:[0,T] \times \mathbb {R_{+}} \rightarrow \mathbb {R_{+}) }$ is said to satisfy the Carathéodory conditions if

$$(C_1)$$
*f*(*t*, *u*) is a continuous function of *u* for almost all $t \in [0,T]$;
$$(C_2)$$
*f*(*t*, *u*) is a measurable function of *t* for all $u \in \mathbb {R}$ (respectively, all $u \in \mathbb {R_{+}}$).

### Definition 2

*f* is said to be an $L^{p}$-Carathéodory function for some $p\in [1,\infty ]$ if it satisfies the Carathéodory conditions and

$(L^{p}-C)$ For each $\rho >0$, there exists $g_{\rho } \in L_{+}^{p}[0,T]$ such that

$$\begin{aligned} |f(t,u)|\le g_{\rho }(t) \text { for a.e.} t \in \text {[0,T]} \text { and all} \ u\in [0,\rho ]. \end{aligned}$$

This class strictly contains the class of continuous functions and, if $p>1/\alpha$, the class of functions *f* having the form $f(t,u)=t^{-\gamma }g(t,u)$ where *g* is continuous and $\gamma <\alpha$, considered in [29].

Given a function *f* satisfying the Carathéodory conditions and a function $u:[0,T] \rightarrow \mathbb {R}$, define a new function $Fu: [0,T] \rightarrow \mathbb {R}$ by

$$\begin{aligned} (Fu)(t)=f(t,u(t)), F \ \text {is called the Nemytskii or substitution operator}. \end{aligned}$$

If *f* is $L^{p}$-Carathéodory, then for $u \in C[0,T]$, $Fu \in L^{p}$.

Since nomenclature is not uniform in the literature we give some definitions. Let *X* be a Banach space with norm $\Vert \cdot \Vert$. Recall that a map, nonlinear or linear, $N:D={\text {dom}}(N)\subset X\rightarrow X$ is said to be bounded if it maps each bounded subset $S\subset D$ into a bounded set. *N* is called compact if $\overline{N(S)}$ (the closure) is a compact set for each bounded subset $S\subset D$. *N* is called completely continuous if *N* is both continuous and compact. Obviously a compact linear operator is bounded and completely continuous.

A cone is a closed convex set $K \subset X$ such that $u,v \in K$ and $\lambda \ge 0$ imply that $u+v \in K$ and ${\lambda }u \in K$, with $K \cap (-K)=\{0\}$.

Let *K* be a cone in *X* both endowed with norm $\Vert x\Vert$. For $r>0$, let ${K}_{r}=\{x\in K: \Vert x\Vert < r\}$, $\overline{K}_{r}=\{x\in K: \Vert x\Vert \le r\}$ and $\partial K_{r}=\{x\in K: \Vert x\Vert =r\}$.

The following well-known result shows that to prove existence of fixed points of a nonlinear map *N* we need two ingredients, a completely continuous operator and an a priori bound.

### Lemma 1

Let $r>0$. If $N:\overline{K}_{r}\rightarrow K$ is completely continuous and satisfies the Leray-Schauder condition:

(*LS*) $u\ne {\lambda }Nu$ for all $u\in \partial K_{r}$ and all $\lambda \in (0,1)$,

then there exists $u\in \overline{K}_{r}$ such that $Nu=u$.

The result is a well-known property of fixed point index when we use a cone *K*, and of Leray-Schauder degree when *K* is replaced by *X* and $K_{r}$ is replaced by the ball $B_{r}:=\{x: \Vert x\Vert <r\}$, etc..

In our case we will obtain compactness from the properties of fractional integrals, as in Sect. 3. The a priori bounds we need will come from new Bihari inequalities which we prove in Sects. 5 and 6.

We often use the elementary inequalities, for $x,y \ge 0$, $m \in [0,\infty )$,

$(x+y)^{m} \le (x^m+y^m)$ if $m \le 1$, and $(x+y)^{m} \le 2^{m-1}(x^m+y^m)$ if $m \ge 1$.

## Fractional integrals and derivatives

In the study of fractional integrals and fractional derivatives, Gamma and Beta functions occur frequently. For $x>0, y>0$, the Gamma function is defined by

3.1 $$\begin{aligned} \Gamma (x):=\int _0^{\infty } s^{x-1}\exp (-s)\,ds, \end{aligned}$$

and the Beta function is defined by

3.2 $$\begin{aligned} B(x,y):=\int _0^1 (1-s)^{x-1}s^{y-1}\,ds. \end{aligned}$$

These are well defined Lebesgue integrals for $x>0, y>0$ and it is well known, and proved in calculus texts, that $B(x,y)=\dfrac{\Gamma (x)\Gamma (y)}{\Gamma (x+y)}$.

### Definition 3

The Riemann-Liouville (R-L) fractional integral of order $\alpha >0$ of a function $f \in L^1[0,T]$ is defined for a.e. *t* by

3.3 $$\begin{aligned} I^{\alpha }f(t):=\frac{1}{\Gamma (\alpha )}\int _0^t (t-s)^{\alpha -1} f(s)\,ds. \end{aligned}$$

The integral $I^{\alpha }u$ is the convolution of the $L^1$ functions *h*, *f* where $h(t)=t^{\alpha -1}/\Gamma (\alpha )$, so by the well known results on convolutions $I^{\alpha }f$ is defined as an $L^1$ function, in particular $I^{\alpha }f(t)$ is finite for a.e. *t*. If $\alpha =1$ this is the usual integration operator which we denote *I*.

Some authors do not state $f \in L^1$ but say the R-L integral exists for a function *f* “provided that the integral exists for $t>0$”; this is not precise, it is not clear which class of functions is being considered, and if it is intended to be for all $t>0$, then it restricts its applicability.

A standard example is the fractional integral of powers of *t*. Consider $h(t)=t^{\gamma }$ for $\gamma >-1$, then $h \in L^1$ and for $0<\alpha <1$, setting $\sigma =ts$ we have

$$\begin{aligned} \begin{aligned} I^{\alpha }h(t)&=\frac{1}{\Gamma (\alpha )}\int ^{t}_{0}(t-s)^{\alpha -1}s^{\gamma }\,ds =\frac{1}{\Gamma (\alpha )}t^{\alpha +\gamma }\int ^{1}_{0}(1-\sigma )^{\alpha -1}\sigma ^{\gamma }\,d\sigma \\&=t^{\alpha +\gamma }\frac{1}{\Gamma (\alpha )}B(\alpha ,1+\gamma )=t^{\alpha +\gamma } \frac{\Gamma (1+\gamma )}{\Gamma (\alpha +\gamma +1)}. \end{aligned} \end{aligned}$$

This shows that $I^{\alpha }h \in L^1$, but, since $\alpha +\gamma$ can be negative, $I^{\alpha }h(t)$ need not exist at $t=0$ and then is not continuous.

We list a few of the useful properties of the fractional integral $I^{\alpha }$ for $0<\alpha <1$, see the texts Diethelm [6], Samko, Kilbas and Marichev (SKM) [25], or the paper [30].

### Proposition 1

Let $$0<\alpha <1$$.

1. The fractional integral operator $I^{\alpha }$ is a bounded linear operator from $L^p[0,T]$ into $L^p[0,T]$ for all $1 \le p \le \infty$.
2. For $1 \le p<1/\alpha$, $I^{\alpha }$ is a bounded linear operator from $L^p[0, T]$ into $L^r[0, T]$ for $1 \le r < p/(1-{\alpha }p)$; if $1<p<1/\alpha$, then $I^{\alpha }$ maps $L^p[0, T]$ into $L^r[0,T]$ for $r={p/(1-{\alpha }p)}$.
3. For $p>1/\alpha$, the operator $I^{\alpha }$ is bounded from $L^p$ into the Hölder space $C^{0,\alpha -1/p}$, thus, for $f \in L^{p}$, $I^{\alpha }f$ is continuous. In particular, $(I^{\alpha }f)(0)=0$.
4. For $p>1/\alpha$, the operator $I^{\alpha }:L^p \rightarrow C[0,T]$ is completely continuous.
5. $I^{\alpha }$ maps *C*[0, *T*] into *C*[0, *T*] and *AC*[0, *T*] into *AC*[0, *T*].
6. $I^{\alpha }$ does not map (all of) $C^1[0,T]$ into $C^1[0,T]$ (e.g. constants).
7. $I^{\alpha }$ does not map (all of) *C*[0, *T*] into *AC*[0, *T*]. (Not simple, see Sect. 9).

The case of equality for *r* in 2 is due to Hardy-Littlewood (HL) [13, §3.3. Theorem 4]. The proof of 3 is due to HL [13, §5.1, Theorem 12]) and is given in SKM [25, Theorem 3.6]. A slightly weaker result with a simpler proof is given in Webb [32, Theorem 4.5]. The compactness result 4 is a consequence of 3 since the Hölder space $C^{0,\alpha -1/p}$ is compactly embedded in *C* (the simple proof of compactness of the embedding is given, for example, in [30, Addendum]). A completely different proof of compactness and continuity is given in Lan [20, Theorem 3.5].

We state another useful property, the semigroup property.

### Lemma 2

Let $\alpha , \beta >0$ and $u \in L^1[0,T]$. Then $I^{\alpha }I^{\beta }u=I^{\alpha +\beta }u$ as $L^1$ functions, thus, $I^{\alpha }I^{\beta }u(t)=(I^{\alpha +\beta }u)(t)$ for a.e. $t \in [0,T]$, in fact for every *t* for which $(I^{\alpha +\beta }|u|)(t)$ exists. If *u* is continuous this holds for all $t\in [0,T]$. If $u \in L^1$ and $\alpha +\beta \ge 1$ equality again holds for all $t\in [0,T]$.

The proof is by interchanging the order of integration, using Fubini’s theorem, for example see the details in [30].

As in SKM [25, Definition 2.3], and KST [15, Eq.(2.1.36)], we will write $I^{\alpha }(L^{1}[0,T])$ to denote the space of functions *f* such that $f=I^{\alpha }g$ for some $g \in L^1$.

The following characterization is known, SKM [25, Theorem 2.3], and is also proved in Lan [18, Proposition 2.2] and Webb [30, Proposition 3.6].

### Proposition 2

Let $\alpha \in (0,1)$ and $f \in L^1[0,T]$. Then $I^{1-\alpha }f \in AC$ and $(I^{1-\alpha }f)(0)=0$ if and only if $f \in I^{\alpha }(L^{1}[0,T])$.

Let *D* denote the usual differentiation operator, $Du=u'$. The Riemann-Liouville (R-L) fractional derivative of order $\alpha \in (0,1)$ is defined as follows.

### Definition 4

For $\alpha \in (0,1)$ and $u \in L^1$ the R-L fractional derivative $D^{\alpha }u$ is defined when $I^{1-\alpha }u\in AC$ by

3.4 $$\begin{aligned} D^{\alpha }u(t):=D\,I^{1-\alpha }u(t), \;\text {a.e.} \; t \in [0,T]. \end{aligned}$$

For $D\,I^{1-\alpha }u(t)$ to be defined for a.e. *t*, it is necessary that $I^{1-\alpha }u$ should be differentiable a.e., but that is not sufficient when considering IVPs for R-L fractional differential equations via a Volterra integral equation. The condition $I^{1-\alpha }u\in AC$ has often been omitted in definitions in published papers, but its necessity was already stated in the monograph SKM, see [25, Definition 2.4] and the related comments in the ‘Notes to §2.6’ [25, p. 83].

### Definition 5

[6, Definition 3.2], [15, p. 91]. The Caputo differential operator is defined by $D_{*}^{\alpha }u:=D^{\alpha }(u-u(0))$ whenever this R-L derivative exists, that is when *u*(0) exists and $I^{1-\alpha }u \in AC$.

Note that $(I^{1-\alpha }u(0))(t) =\frac{u(0)t^{\alpha }}{\Gamma (\alpha +1)}\in AC$, so the condition $I^{1-\alpha }u \in AC$ is the same as $I^{1-\alpha }(u-u(0)) \in AC$.

Another often used definition of Caputo derivative is when the derivative and fractional integral are taken in the reverse order to that taken in the R-L derivative.

### Definition 6

For $\alpha \in (0,1)$ and $u \in AC$ the Caputo fractional derivative $D_{C}^{\alpha }u$ is defined for a.e. *t* by $D_{C}^{\alpha }u(t):=I^{1-\alpha }Du(t)=I^{1-\alpha }u'(t)$.

For $u \in AC$, $Du \in L^1$ and so $D_{C}^{\alpha }u=I^{1-\alpha }(Du)$ is defined as an $L^1$ function. Unfortunately, this definition has severe disadvantages which make its use questionable, see Sect. 9 for more details.

## Equivalence of IVP and integral equation

We will study the existence of solutions of initial value problems (IVPs) for fractional differential equations (FDEs) of order $\alpha \in (0,1)$ of the form

4.1 $$\begin{aligned} D_{*}^{\alpha }u(t)=f(t,u(t))\;\text {for a.e.} \; t \in [0,T], \;\;u(0)=u_{0}, \end{aligned}$$

where $u_{0}$ is given, and when *f* is $L^{p}$-Carathéodory.

### Definition 7

Let *f* be $L^{p}$-Carathéodory. A solution of the IVP (4.1) on an interval [0, *T*] is a continuous function *u* with $u(0)=u_{0}$, $I^{1-\alpha }u \in AC$, and such that $D(I^{1-\alpha }(u-u_{0}))(t)=f(t,u(t))$ for a.e. $t \in [0,T]$.

Let *P* denote the standard cone of non-negative continuous functions, that is,

4.2 $$\begin{aligned} P=\{u\in C[0,T]: u(t)\ge 0\; \text {for each} \; t \in [0,T]\}. \end{aligned}$$

*P* inherits the norm of *C*[0, *T*].

Since there is almost no difference between the arguments for existence of solutions in *C*[0, *T*] and the existence of non-negative solutions in *P*, we will concentrate on the study of non-negative solutions when $f(t,u) \ge 0$ for $t \in [0,T]$ and $u \ge 0$.

We will study non-negative solutions of (4.1) via fixed points in the cone *P* of the nonlinear integral operator *N* defined by

4.3 $$\begin{aligned} Nu(t)=u_{0}+(I^{\alpha }Fu)(t) \;\text {for each} \;t \in [0,T], \end{aligned}$$

where *F* is the Nemytskii operator $(Fu)(t)=f(t,u(t))$.

We will prove equivalence of these problems in Lemma 4. First we prove an important property of the Nemytskii operator.

### Lemma 3

Let $f:[0,T]\times \mathbb R_{+}\rightarrow \mathbb R_{+}$ be $L^{p}$-Carathéodory for some $p\in [1,\infty )$. Then the Nemytskii operator *F* maps *P* into $L_{+}^{p}$ and is bounded and continuous.

### Proof

Let $u\in P$. By $(L^{p}-C)$, $Fu\in L_{+}^{p}$, hence, *F* maps *P* into $L_{+}^{p}$. Let $S\subset P$ be bounded. Then there exists $\rho >0$ such that $\Vert u\Vert _{\infty }\le \rho$ for every $u\in S$. By $(L^{p}-C)$, we have

$$\begin{aligned} \int _{0}^{T}|(Fu)(t)|^{p}\,dt=\int _{0}^{T}|f(t,u(t))|^{p}\,dt\le \int _{0}^{T}|g_{\rho }(t)|^{p}\,dt, \; \text {for all} \; u\in S, \end{aligned}$$

thus $F:P\rightarrow L_{+}^{p}(0,T)$ is bounded. Suppose that $u_{n} \rightarrow u$ in *P*. Then there exists $\rho >0$ such that $\Vert u_{n}\Vert _{\infty }\le \rho$ and $\Vert u\Vert _{\infty }\le \rho$. By the Carathéodory conditions $u \mapsto f(t,u)$ is continuous for a.e. $t \in [0,T]$ and we have

$$\begin{aligned} \lim _{n\rightarrow \infty }|f(t,u_{n}(t))-f(t,u(t))|^{p}=0\; \text {for a.e} \; t \in [0,T]. \end{aligned}$$

By $(L^{p}-C)$, we have for a.e. $t \in [0,T]$,

$$\begin{aligned} f(t,u_{n}(t))\le g_{\rho }(t) \; \text {and} \;f(t,u(t)) \le g_{\rho }(t). \end{aligned}$$

This implies that

$$\begin{aligned} |f(t,u_{n}(t))-f(t,u(t))|^{p}\le \bigl (f(t,u_{n}(t))+f(t,u(t))\bigr )^{p}\le 2^{p}g_{\rho }^{p}(t),\, \text {a.e.} \; t \in [0,T]. \end{aligned}$$

Since $g_{\rho } \in L^{p}$, by Lebesgue’s dominated convergence theorem we have

$$\begin{aligned} \lim _{n\rightarrow \infty }\int _{0}^{T}|(Fu_{n})(t)-(Fu)(t)|^{p}\,dt=\int _{0}^{T}\lim _{n\rightarrow \infty }|f(t,u_{n}(t))-f(t,u(t))|^{p}\,dt=0, \end{aligned}$$

which proves that $F:P\rightarrow L_{+}^{p}$ is continuous. $\square$

We now show the equivalence between solutions of (4.1) and fixed points of *N* in (4.3), which will apply to *Fu* when *u* is continuous and *f* is $L^{p}$-Carathédory for $p>1/\alpha$, the case we study. The result is very similar to Theorem 4.2 in [18]. For completeness, we provide a proof.

### Lemma 4

Let $0<\alpha <1$ and suppose that $f \in L^{p}$ for some $p>1/\alpha$. Then the following assertions are equivalent.

(*i*) $$u \in C[0,T]$$ satisfies

4.4 $$\begin{aligned} u(t)=u_{0}+I^{\alpha }f(t)\; \text {for each}\; t\in [0,T]. \end{aligned}$$

(*ii*) $u \in C[0,T]$, $I^{1-\alpha } u \in AC$, and *u* is a solution of the IVP

4.5 $$\begin{aligned} D_{*}^{\alpha }u(t)=f(t)\; \text {for a.e.}\; t \in [0,T], \;u(0)=u_{0}. \end{aligned}$$

### Proof

Suppose that (*i*) holds. For $p>1/\alpha$ and $f\in L^{p}[0,T]$, we have $I^{\alpha }f \in C[0,T]$ (Proposition 1 (2)) and so $u(0)=u_{0}$. Using the semigroup property given in Lemma 2, we get $I^{1-\alpha }(u-u_{0})(t)=If(t)$ which is valid for every $t \in [0,T]$ since $I^{1-\alpha }u$ is continuous and $If \in AC$. Thus $I^{1-\alpha }(u-u_{0}) \in AC$ and differentiating gives $D_{*}^{\alpha }u(t)=f(t)\; \text {for a.e.}\; t \in [0,T]$.

Now let (*ii*) be satisfied. Then $D_{*}^{\alpha }u(t)=D(I^{1-\alpha }(u-u_{0}))(t)=f(t)$ a.e. and $I^{1-\alpha }(u-u_{0})(0)=0$. Using the AC property, we can integrate to obtain $I^{1-\alpha }(u-u_{0})(t)=If(t)$. Applying $I^{\alpha }$ and using the semigroup property proves $I(u-u_{0})(t)=I\,I^{\alpha }f(t)$, differentiating gives $u(t)-u_{0}=I^{\alpha }f(t)$ for every $t \in [0,T]$, since both sides are continuous functions. $\square$

## New Bihari inequality for regular kernels

### Theory

The Bihari inequality was proved by Bihari in [1]. It was assumed that a non-negative continuous function *u* satisfies the inequality

5.1 $$\begin{aligned} u (t) \le a + b\int _{0}^{t} \phi (s) w(u(s)) \,ds, \;\text {for}\;t \in [0,T], \end{aligned}$$

where *a*, *b* are positive constants, $\phi$ is continuous and non-negative, $w:[0, \infty ) \rightarrow [0,\infty )$ is a non-negative non-decreasing continuous function and there exists $0<x_0 \le a$ such that $w(x_0)>0$. Then for $x_{0} \le x_{1} \le a$ a function *W* is defined by

5.2 $$\begin{aligned} W(x)=\int _{x_{1}}^{x}{\frac{dy}{w(y)}},\;\; x\ge x_{1}, \end{aligned}$$

and it is deduced that

5.3 $$\begin{aligned} u(t)\le W^{-1}\Bigl (W(a)+b\int _{0}^{t}\,\phi (s)\,ds\Bigr ),\; t\in [0,T_1], \end{aligned}$$

where $T_{1}$ is such that the *range condition*

5.4 $$\begin{aligned} W_{a,b,\phi }(t):=W(a)+b\int _{0}^{t}\,\phi (s)\,ds \in {\text {range}}(W), \;\text {for all} \;t \in [0,T_1], \end{aligned}$$

holds. Since *W* is a strictly increasing continuous function, $W(x) \rightarrow W_{\infty }$ as $x \rightarrow \infty$ (where $W_{\infty }=\infty$ is allowed) and ${\text {range}}(W)=[0,W_{\infty })$, hence its inverse $W^{-1}$ is well defined with ${\text {dom}}(W^{-1})={\text {range}}(W)$ and $W^{-1}:[0,W_{\infty }) \rightarrow [x_{1}, \infty )$ is a continuous, strictly increasing function. The range condition can be written $0 \le W_{a,b,\phi }(t) <W_{\infty }$ for all $t \in [0,T_1]$. Since $W(a) \ge W(x_{1})=0$, $T_{1} \ge 0$ always exists. In the trivial case where $\phi (t)=0$ for a.e. *t*, $W_{a,b,\phi }(t)=W(a) \in {\text {range}}(W)$ for all *t* and then $T_{1}=T$. From (5.1) we get $u(t) \le a$; the conclusion (5.3) is correct, but no Bihari inequality is required in this case. We suppose this trivial case does not occur. Note that, either $W_{\infty }=\infty$ and so (5.4) always holds and $T_1=T$, or else $W_{\infty }<\infty$, then either there exists $T_0 \le T$ such that $W_{a,b,\phi }(T_0)=W_{\infty }$ so that $T_1<T$, or $W_{a,b,\phi }(T)<W_{\infty }$ which gives $T_1=T$ and (5.3) holds for all $t \in [0,T]$.

We need a more general version when $\phi \in L^1_{+}$ and *a*, *b* can be functions in (5.1) where it is supposed that $a(t) \ge x_{1} \ge x_{0}$.

**Notation** For bounded functions $a,b:[0,T] \rightarrow \mathbb {R}_{+}$ we denote by *A*, *B* the functions $A(t):=\sup _{s \in [0,t]}a(s)$, $B(t):=\sup _{s \in [0,t]}b(s)$.

We use the notations as given above and we write:

5.5 $$\begin{aligned} W_{A,B,\phi }(t):=W(A(t))+B(t)\int _{0}^{t}\,\phi (s)\,ds. \end{aligned}$$

Since *A*, *B* are non-decreasing, $W_{A,B,\phi }$ is also non-decreasing and $W_{A,B,\phi }(0)=W(a(0))$ exists and is non-negative since it will be assumed that $a(t) \ge x_{1} \ge x_{0}$. $W_{A,B,\phi }(t)$ will be positive except in the trivial case where $W(A(t))=0$ and $B(t)\int _{0}^{t} \phi (s)\,ds=0$. We will exclude this trivial case.

#### Theorem 1

Let *a*, *b* be positive, bounded functions, let $u \in C_{+}[0,T]$, $\phi \in L^1_{+}[0,T]$ with $\phi >0$ on a set of positive measure, and let *w* be a non-negative, continuous, non-decreasing function defined on $[0, \infty )$ and $w(x_0) > 0$ for some $x_0>0$. Suppose that $a(t) \ge x_{1} \ge x_0$ for every $t \in [0,T]$ and *u* satisfies the integral inequality,

5.6 $$\begin{aligned} u (t) \le a(t) + b(t)\int _{0}^{t} \phi (s) w(u(s)) \,ds, \;\text {for}\;t \in [0,T]. \end{aligned}$$

Then, for *W* defined by $W(x)=\int _{x_{1}}^{x}{\frac{dy}{w(y)}}\;\text {for}\; x\ge x_{1}$, we have

5.7 $$\begin{aligned} u(t)\le W^{-1}\Bigl (W(A(t))+B(t)\int _{0}^{t}\,\phi (s)\,ds\Bigr )=W^{-1}\bigl (W_{A,B,\phi }(t)\bigr ), \end{aligned}$$

for every $t\in [0,T_1]$, where $T_{1}$ is such that

5.8 $$\begin{aligned} W_{A,B,\phi }(t) \in {\text {range}}(W)\;\text {for all} \;t \in [0,T_1], \;\text {that is,}\;0 \le W_{A,B,\phi }(T_{1})<W_{\infty }. \end{aligned}$$

#### Proof

We first consider the case when *a*, *b* are positive constants. Define a function *v* by

$$\begin{aligned} v(t)=a + b\int _{0}^{t} \phi (s) w(u(s)) \,ds. \end{aligned}$$

Then $v(0)=a$, $v(t)\ge a \ge x_0 >0$, $u(t) \le v(t)$ and $v \in AC[0,T]$ since *w*(*u*) is continuous and $\phi \in L^1$. Hence $v'(t)$ exists for a.e. *t* and is an $L^1$ function. Since *w* is non-decreasing, we have for a.e. *t*,

$$\begin{aligned} v'(t)=b\phi (t)w(u(t)) \le b\phi (t)w(v(t)), \;\text {or}\;\frac{v'(t)}{w(v(t))} \le b\phi (t), \;\text {a.e.}\; t, \end{aligned}$$

where $w(v(t))>0$ for every $t \in [0,T]$. Note that *W* is a strictly increasing $C^1$ function, hence is Lipschitz on every bounded interval, therefore $W(v)\in AC[0,T]$. By integration we obtain

$$\begin{aligned} W(v(t))-W(v(0)) \le b\int _0^t \phi (s)\,ds, \;\text {that is}\;\; W(v(t)) \le W_{a,b,\phi }(t). \end{aligned}$$

By the range condition (5.8) and since $W^{-1}$ is increasing, we deduce that

5.9 $$\begin{aligned} u(t) \le v(t) \le W^{-1}\bigl (W_{a,b,\phi }(t)\bigr ), \;\text {for}\; t\in [0,T_{1}]. \end{aligned}$$

Secondly, suppose that *a*, *b* are positive functions with $a(t) \ge x_{1}$. For $\tau \in [0,T_1]$ (arbitrary) we have

$$\begin{aligned} u (t) \le A(\tau ) + B(\tau )\int _{0}^{t} \phi (s) w(u(s)) \,ds, \;\text {for}\;t \in [0,\tau ]. \end{aligned}$$

By the assumption (5.8), for all $t \in [0,\tau ]$,

$$\begin{aligned} W(A(\tau )) + B(\tau )\int _{0}^{t} \phi (s) \,ds \le W(A(\tau )) + B(\tau )\int _{0}^{\tau } \phi (s) \,ds <W_{\infty }. \end{aligned}$$

Thus, by the case just proved, this gives

$$\begin{aligned} u(t) \le W^{-1}\Bigl (W(A(\tau ))+B(\tau )\int _0^t \phi (s)\,ds\Bigr ), \;\text {for}\; t\in [0,\tau ]. \end{aligned}$$

This inequality holds for $t=\tau$, so, since $\tau \in [0,T_1]$ is arbitrary, (5.7) holds. $\square$

#### Remark 1

If $w(u)>0$ for all $u>0$ then we may assume that *a* is only non-negative by taking $x_{0}$ arbitrarily small, replacing *a* by $a+x_{0}$ and then let $x_{0} \rightarrow 0$. Of course $A(t)=a(t)$ and $B(t)=b(t)$ when *a* and *b* are non-decreasing functions. It is necessary that $x_{1} \le a$ so that $W(a) \ge 0$ exists according to our definition. We allow $w(0)=0$, but it is only required that $w(0) \ge 0$ and $w(x_{0})>0$ for some $x_{0}>0$.

The original result was proved by Bihari who had *a*, *b* constants and supposed that $\phi$ is continuous, but it was only implicit that $w(x_{0})>0$ for some $x_{0}>0$. Bihari showed that although the definition of *W* depends on $x_{1}>0$ the final estimate (5.9) does not depend on $x_{1}$. Since this can easily be mis-stated we give a precise result for the case when *a*, *b* are constants.

#### Theorem 2

Let the hypotheses of Theorem 1 hold. Suppose there are two possible initial points $x_{1},x_{2}$, with $x_{0} \le x_{1} \le a$ and $x_{2}>x_{1}$, with possibly $x_{2}>a$, and define $W_{1}, W_{2}$ with a *common domain of definition* $[x_{1},\infty )$ by

5.10 $$\begin{aligned} W_{j}(x):=\int _{x_{j}}^x \frac{du}{w(u)}, \;x \ge x_{1},\;j=1,2. \end{aligned}$$

Then we have

5.11 $$\begin{aligned} W_{2}^{-1}\Bigl (W_{2}(a)+b\int _0^t \phi (s)\,ds\Bigr )=W_{1}^{-1}\Bigl (W_{1}(a)+b\int _0^t \phi (s)\,ds\Bigr ). \end{aligned}$$

#### Proof

Note that $W_{2}(x)$ can take negative values when $x_{1} \le x < x_{2}$. In fact, thanks to the common domain of definition we have

$$\begin{aligned} W_{1}(x):=\int _{x_{1}}^x \frac{du}{w(u)}=\int _{x_{1}}^{x_{2}} \frac{du}{w(u)}+\int _{x_{2}}^x \frac{du}{w(u)} =:\delta +W_{2}(x), \;\text {for}\; x \ge x_{1}, \end{aligned}$$

where $\delta$ is a positive constant. Then ${\text {range}}(W_{1})=[0,c)$ implies that ${\text {range}}(W_{2})=[-\delta ,c-\delta )$ and $W_{2}(a)+b\int _0^t \phi (s)\,ds \in [-\delta ,c-\delta )$ if and only if $W_{1}(a)+b\int _0^t \phi (s)\,ds \in [0,c)$. Moreover $W_{2}^{-1}(y)=W_{1}^{-1}(y+\delta )$ for $y \in [-\delta ,c-\delta )$. Using this easily gives the equality (5.11). $\square$

#### Remark 2

The common domain of definition is needed otherwise $W_{2}(a)$ might not be defined. The same result will apply to the case of functions since the proof in Theorem 1 reduces it to the case of constants.

Butler and Rogers [2] gave a more general version of the Bihari inequality when $\phi (s)$ is replaced by *k*(*t*, *s*) and *u* is replaced by *f*(*u*) for suitable functions *f*, *k*. They considered the case when *a*, *b* are positive functions by reduction to the case when *a*, *b* are constants using the same method as in the proof above. They did not check that certain functions are *AC* so that the integration step was not shown to be valid, some extra conditions on their functions are required to justify this.

We have not been able to find the results of Theorems 1 and 2 in the literature when $\phi \in L^1$; we welcome any information. The method of the proof is the standard one but with some extra care needed.

### Examples

We first consider three standard examples when $w(u)=u^r$ for $r>0$, for $0<r<1$, for $r=1$, and for $r>1$. The first two cases give global estimates valid for arbitrary *T*, the third only gives a local estimate. We then give a fourth family of examples with $w_{k}(u)=(k+u)\ln (k+u)$ where $k \ge 1$ and again we get a global estimate valid for arbitrary *T*. These are cases where *w*(*u*) grows faster than linear, but to be valid for any *T*, such a function *w* must grow more slowly than $u^{r}$ for any power $r>1$.

In all the examples it is supposed that the hypotheses and notation of Theorem 1 hold.

#### Example 1

$w(u)=u^r$ for $0<r<1$. This case is well-known when *a*, *b* are constants and is easy to calculate, we only state the result. In this case ${\text {range}}(W)=[0,\infty )$ and the range condition is always satisfied. The result is that if

5.12 $$\begin{aligned} u(t) \le a(t)+b(t) \int _0^t \phi (s)u^{r}(s)\,ds, \;t \in [0,T], \end{aligned}$$

holds for an arbitrary $T>0$, then, from Theorem 1,

5.13 $$\begin{aligned} u(t) \le \Bigl (A(t)^{1-r}+(1-r)B(t)\int _0^t \phi (s)\,ds\Bigr )^{1/(1-r)}, \;\text {for all} \;t \in [0,T]. \end{aligned}$$

#### Example 2

$w(u)=u$, the Gronwall inequality. Since the final estimate does not depend on the initial point $x_{0}$, we choose $x_{1}=1$ and let $W(x)=\int _1^x \frac{1}{u}du=\ln (x)$. Then ${\text {range}}(W)=[0,\infty )$ so the range condition is always satisfied and $W^{-1}(y)=\exp (y)$, $y \ge 0$. Let $u \ge 0$ be continuous and satisfy the inequality

5.14 $$\begin{aligned} u(t) \le a(t)+b(t)\int _{0}^{t}\phi (s)u(s)\,ds, \;\text {for} \;t \in [0,T], \end{aligned}$$

for an arbitrary $T>0$. Then we have

5.15 $$\begin{aligned} u(t) \le A(t)\exp \Bigl (B(t)\int _{0}^{t} \phi (s)\,ds\Bigr ), \;\text {for} \;t \in [0,T]. \end{aligned}$$

The easy calculation is omitted.

#### Example 3

$w(u)=u^r$ for $r>1$. This case is known when *a*, *b* are constants and $\phi$ is continuous, we give some details to illustrate that the bound obtained does not depend on the choice of initial point $x_{0}$. The result is as follows. If

5.16 $$\begin{aligned} u(t) \le a+b \int _0^t \phi (s)u^{r}(s)\,ds, \;t \in [0,T], \end{aligned}$$

then

5.17 $$\begin{aligned} u(t) \le \frac{1}{\Bigl (\frac{1}{a^{r-1}}-b(r-1)\int _0^t \phi (s)\,ds\Bigr )^{1/(r-1)}}, \;t \in [0,T_1], \end{aligned}$$

where $T_{1}$ is such that $a^{r-1}b(r-1)\int _0^{T_1}\phi (s)\,ds <1$.

In fact, let $0<x_{0}\le a$ and let

$$\begin{aligned} W(x):=\int _{x_{0}}^x \frac{du}{u^r}=\frac{1}{r-1}\Bigl [\frac{1}{x^{r-1}_{0}}-\frac{1}{x^{r-1}}\Bigr ]. \end{aligned}$$

Then ${\text {range}}(W)=[0, \frac{1}{(r-1)}\frac{1}{x^{r-1}_{0}})$, and for $y \in {\text {range}}(W)$,

$$\begin{aligned} W^{-1}(y)=\dfrac{x_{0}}{\bigl (1-x_{0}^{r-1}(r-1)y\bigr )^{1/(r-1)}}. \end{aligned}$$

Then we obtain

5.18 $$\begin{aligned} \begin{aligned}&W^{-1}\bigl (W(a)+b\int _{0}^t\phi (s)\,ds\bigr )\\&=\frac{x_{0}}{\Bigl (1-x_{0}^{r-1}(r-1)\Bigl [\frac{1}{r-1}\bigl (\frac{1}{x^{r-1}_{0}}-\frac{1}{a^{r-1}}\bigr ) + b\int _{0}^t\phi (s)\,ds\Bigr ]\Bigr )^{1/(r-1)}}. \end{aligned} \end{aligned}$$

Simplifying, the $x_{0}$ term on upper and lower part of the fraction cancels, and we get the final result (5.17).

In this case, for a given *T*, it can happen that $T_{1}=T$, for example when *a*, *b* and $\int _0^{T} \phi (s)\,ds$ are sufficiently small, but the result will usually not apply for an arbitrary $T>0$.

#### Example 4

Let $w(u)=(1+u)\ln (1+u)$ so that *w* is positive and increasing for $u>0$. Suppose $u \ge 0$ is continuous and satisfies the inequality

5.19 $$\begin{aligned} u (t) \le a + b\int _{0}^{t} \phi (s) w(u(s)) \,ds, \;\text {for}\;t \in [0,T], \end{aligned}$$

for an arbitrary $T>0$. Let $0<x_0 \le a$ and define

$$\begin{aligned} W(x)=\int _{x_{0}}^{x} \frac{du}{w(u)}=\ln (\ln (1+x))-\ln (\ln (1+x_{0})), \;\text {with}\; {\text {dom}}(W)=[x_0,\infty ). \end{aligned}$$

Then *u* satisfies the bound

5.20 $$\begin{aligned} u(t)\le (1+a)^{\exp (b\int _0^t \phi (s)\,ds)}-1,\;\text {for all}\; t\in [0,T]. \end{aligned}$$

We show how the Bihari inequality applies. Since the final estimate does not depend on the initial point $x_{0}$, we choose $x_{1}=\exp (1)-1$ and consider $W_{1}(x)=\int _{x_{1}}^{x} \frac{du}{w(u)}=\ln (\ln (1+x))$. Then the range condition is satisfied and $W_{1}^{-1}(y)=\exp (\exp (y))-1$ for $y \ge 0$ and from Theorem 1, after some simplification, we obtain the conclusion

5.21 $$\begin{aligned} u(t)\le W_{1}^{-1}\Bigl (W_{1}(a)+b\int _{0}^{t}\,\phi (s)\,ds\Bigr )=(1+a)^{\exp (b\int _0^t \phi (s)\,ds)}-1, \end{aligned}$$

for all $$t\in [0,T]$$.

Similarly, we can consider $w_{k}(u)=(k+u)\ln (k+u)$ for any constant $k \ge 1$. From the inequality

5.22 $$\begin{aligned} u (t) \le a + b\int _{0}^{t} \phi (s) w_{k}(u(s)) \,ds, \;\text {for}\;t \in [0,T], \end{aligned}$$

by essentially the same calculation we obtain the conclusion

5.23 $$\begin{aligned} u(t)\le (k+a)^{\exp (b\int _0^t \phi (s)\,ds)}-k,\;\text {for all}\; t\in [0,T]. \end{aligned}$$

We can replace the constants *a*, *b* by functions *A*, *B* as in Theorem 1. The inequality (5.23) is an explicit a priori bound, which is valid for every $T>0$.

#### Remark 3

A slightly less general result than that of Example 4 was proved, with another notation, in Engler [11, p.257] by a direct integration, which improved on a result in Haraux [12, p.139]. Engler does not give the case when the constants are replaced by functions. We believe this is the first time the result has been shown as a corollary of Bihari’s inequality.

## New Bihari inequality for singular kernels

To obtain a priori bounds for the fractional integral equation

6.1 $$\begin{aligned} u(t)=u_{0}+\int _{0}^{t} (t-s)^{\alpha -1}f(s,u(s))\,ds, \end{aligned}$$

when *f* is $L^{p}$-Carathéodory and satisfies some growth condition, we will apply a suitable Bihari inequality. The following new result fits our needs.

### Theorem 3

Let $0<\alpha <1$, $1/\alpha<p<\infty$ and $q=p/(p-1)$. Let $u \in C_{+}[0,T]$, $\phi \in L^{p}_{+}[0,T]$ with $\phi >0$ on a set of positive measure, and let $\widehat{w}:[0,\infty )\rightarrow [0,\infty )$ be a function such that $\widehat{w}^{p}(u) \le w(u^{p})$ for all $u>0$, where *w* is a non-decreasing continuous function and $w(x_0)>0$ for some $x_{0}>0$. Let *a*, *b* be positive bounded functions such that $2^{p-1}a^{p}(t) \ge x_{0}$ for $t \in [0,T]$ and suppose that

6.2 $$\begin{aligned} u(t)\le a(t)+b(t)\int _0^{t}(t-s)^{\alpha -1}\phi (s) \widehat{w}(u(s))\,ds, \;\text {for}\; t\in [0,T]. \end{aligned}$$

Define $W(x):=\int ^{x}_{x_{0}}\frac{dy}{w(y)}$. Then we have

6.3 $$\begin{aligned} u(t) \le \Bigl (W^{-1}\bigl [W(2^{p-1}A^{p}(t))+2^{p-1}C(t)\int _0^t \phi ^{p}(s)\,ds\bigr ]\Bigr )^{1/p}, \;\text {for}\; t\in [0,T_{1}]\nonumber \\ \end{aligned}$$

where $T_{1}$ is such that the following range condition holds

6.4 $$\begin{aligned} W(2^{p-1}A^{p}(t))+2^{p-1}C(t)\int _0^t \phi ^{p}(s)\,ds\in {\text {range}}(W), \;\text {for}\; t \in [0,T_{1}], \end{aligned}$$

where

6.5 $$\begin{aligned} C(t):=B^{p}(t)\Bigl (\frac{t^{q(\alpha -1)+1}}{q(\alpha -1)+1}\Bigr )^{p-1}. \end{aligned}$$

### Proof

Applying Hölder’s inequality in (6.2) gives

$$\begin{aligned} u(t)\le a(t)+b(t)\Bigl (\int _0^{t}(t-s)^{q(\alpha -1)}\,ds\Bigr )^{1/q} \Bigl (\int _0^t \phi ^{p}(s) \widehat{w}^{p}(u(s))\,ds\Bigr )^{1/p}. \end{aligned}$$

Note that $q(\alpha -1)>-1$ since we are taking $p>1/\alpha$, so the first term is integrable. Thus we obtain

$$\begin{aligned} u(t)\le a(t)+b(t)\Bigl (\frac{t^{q(\alpha -1)+1}}{q(\alpha -1)+1}\Bigr )^{1/q} \Bigl (\int _0^t \phi ^{p}(s) w(u^{p}(s))\,ds\Bigr )^{1/p}. \end{aligned}$$

Raising both sides to the power *p* and using the inequality $(x+y)^{p} \le 2^{p-1}(x^p+y^p)$, ($x,y \ge 0$, $p \ge 1$), we obtain

$$\begin{aligned} u^{p}(t)&\le 2^{p-1}a^{p}(t)+2^{p-1}b^{p}(t)\Bigl (\frac{t^{q(\alpha -1)+1}}{q(\alpha -1)+1}\Bigr )^{p/q} \Bigl (\int _0^t \phi ^{p}(s) w(u^{p}(s))\,ds\Bigr )\\&=2^{p-1}a^{p}(t)+2^{p-1}c(t) \Bigl (\int _0^t \phi ^{p}(s) w(u^{p}(s))\,ds\Bigr ), \end{aligned}$$

where

$$\begin{aligned} c(t):=b^{p}(t)\Bigl (\frac{t^{q(\alpha -1)+1}}{q(\alpha -1)+1}\Bigr )^{p-1}. \end{aligned}$$

This is now the Bihari inequality of Theorem 1. Hence we deduce that

$$\begin{aligned} u^{p}(t)\le W^{-1}\bigl [W(2^{p-1}A^{p}(t)\bigr )+2^{p-1}C(t)\int _0^t \phi ^{p}(s)\,ds\bigr ], \;\text {for}\; t\in [0,T_{1}], \end{aligned}$$

with $C(t)=\sup _{0 \le s \le t}c(s)$ is as given in (6.5). This gives (6.3). $\square$

### Remark 4

When $\widehat{w}$ is continuous and non-decreasing the optimal choice of *w* is to take $w(v)=\widehat{w}^{p}(v^{1/p})$. However it is possible to apply simple inequalities to get a suitable *w* when $\widehat{w}$ is not non-decreasing; see Remark 6 below.

### Remark 5

This gives a result similar to Zhu [33, Theorem 3.1], who had $\widehat{w}(u)=u$. Our result is more general and we also have a simpler form of conclusion.

Our result generalizes and corrects the similar result of [24, Theorem 2.3], in which *a* is a constant, $b=1$, and it is assumed that $\phi$ is continuous, $\widehat{w}$ is non-decreasing and $\widehat{w}(0)=0$. The statement of Theorem 2.3 in [24] does not give the important domain condition without which the result is not correct, and it is stated that the final inequality holds on [0, *T*] which is false in general. The proof uses Hölder’s inequality, as above, and then essentially gives the proof of the original Bihari inequality. In the equations (2.3), (2.4) of [24], there are actually two different functions both called $\Psi$. There are also several minor typos.

### Remark 6

For the examples $\widehat{w}(u)=u^{r}$ with $r>0$ we have $w=\widehat{w}$ and we can apply the results of Examples 1,2,3. A new family of examples is $\widehat{w}_{k}(u)=(k^{1/p}+u)(\ln (k+u^{p}))^{1/p}$ for $k \ge 1$. Then $\widehat{w}^{p}_{k}(u) \le w_{k}(u^p)$ for $w_{k}(v)=2^{p-1}(k+v)\ln (k+v)$ where we can use Example 4.

An example where $\widehat{w}$ is not increasing, but *w* can be easily given, is

$$\begin{aligned} \widehat{w}(u)= (1+u+3\sin ^2(u))(\ln (1+u^{p}))^{1/p}. \end{aligned}$$

$\widehat{w}$ is not an increasing function but we have

$$\begin{aligned} \widehat{w}^{p}(u)\le (4+u)^{p}\ln (1+u^{p})< 2^{p-1} (4^{p}+u^{p})\ln (4^{p}+u^{p}) \end{aligned}$$

and we may take $w(v)=2^{p-1} (4^{p}+v)\ln (4^{p}+v)$ and use Example 4.

## Global existence results

We will work in the space $C[0,T_{1}]$ for suitable $T_{1} \le T$. In particular we study non-negative solutions of the IVP

7.1 $$\begin{aligned} D_{*}^{\alpha }u(t)=f(t,u(t))\;\text {for a.e.} \; t \in [0,T_{1}], \;\;u(0)=u_{0}, \end{aligned}$$

where $\alpha \in (0,1)$, $u_{0}\in \mathbb {R}_{+}$ is given.

### Theorem 4

Assume that $u_{0}\in \mathbb {R}_{+}$, $f \ge 0$ is $L^{p}$-Carathéodory for $p>1/\alpha$ and there exist $\xi ,\phi \in L_{+}^{p}[0,T]$ with $\int _{0}^{T}\phi ^{p}(s)\,ds>0$, such that

7.2 $$\begin{aligned} f(t,u)\le \xi (t)+\phi (t)\widehat{w}(u)\; \text {for a.e.}\; t\in [0,T]\; \text {and}\; u\in \mathbb {R}_{+}, \end{aligned}$$

where $\widehat{w}:[0,\infty )\rightarrow [0,\infty )$ is such that $\widehat{w}^{p}(u) \le w(u^{p})$ for all $u>0$, where *w* is a non-decreasing continuous function with $w(x_0)>0$ for some $x_{0}>0$. Then (7.1) has a solution $u\in C_{+}[0,T_{1}]$, where $T_{1} \le T$ is given in (7.4) below.

### Proof

Let $N:P \rightarrow C[0,T]$ be defined by

$$\begin{aligned} Nu(t):=u_{0}+I^{\alpha }Fu=u_{0}+\frac{1}{\Gamma (\alpha )}\int _{0}^{t} (t-s)^{\alpha -1}Fu(s)\,ds, \end{aligned}$$

where *F* is the Nemytskii operator. As *f* is $L^{p}$-Carathéodory, *F* is continuous from *C*[0, *T*] into $L^{p}[0,T]$ by Lemma 3 and $I^{\alpha }$ is completely continuous from $L^{p}[0,T]$ into *C*[0, *T*] by Proposition 1 4, so *N* is completely continuous. Now suppose that $u(t)=\lambda Nu(t)$ for some $\lambda \in [0,1]$. Then we have

$$\begin{aligned} \begin{aligned} u(t)=\lambda Nu(t)&\le Nu(t)\le u_{0}+\frac{1}{\Gamma (\alpha )}\Bigl (\int _0^{t} (t-s)^{\alpha -1}\bigl (\xi (s)+\phi (s)\widehat{w}(u(s))\bigr )\,ds\Bigr )\\&= u_{0}+I^{\alpha }\xi (t)+\frac{1}{\Gamma (\alpha )}\Bigl (\int _0^{t} (t-s)^{\alpha -1}\phi (s)\widehat{w}(u(s))\,ds\Bigr ). \end{aligned} \end{aligned}$$

Noting that $I^{\alpha }\xi$ is continuous (Proposition 1, 3), hence bounded on [0, *T*], this is exactly the situation of Theorem 3 with $a(t)= u_{0}+I^{\alpha }\xi (t)$, $b=\frac{1}{\Gamma (\alpha )}$. Therefore we can conclude that

7.3 $$\begin{aligned} u(t) \le \Bigl (W^{-1}\bigl [W(2^{p-1}A^{p}(t))+2^{p-1}C(t)\int _0^t \phi ^{p}(s)\,ds\bigr ]\Bigr )^{1/p}, \;\text {for}\; t\in [0,T_{1}],\nonumber \\ \end{aligned}$$

where $W(x):=\int _{x_{0}}^{x}\frac{du}{w(u)}$, and $C(t)=\bigl (\frac{1}{\Gamma (\alpha )}\bigr )^{p}\bigl [\frac{t^{q(\alpha -1)+1}}{q(\alpha -1)+1}\bigr ]^{p-1}$, and $T_{1}$ is such that

7.4 $$\begin{aligned} W(2^{p-1}A^{p}(t))+2^{p-1}C(t)\int _0^t \phi ^{p}(s)\,ds\in {\text {range}}(W), \;\text {for every}\; t \in [0,T_{1}]. \end{aligned}$$

We have shown that, for $T_{1}$ as in (7.4), there is a constant $M=M(T_{1})$ such that if $u={\lambda } Nu$ then $u(t) \le M$ for $0 \le t \le T_{1}$. Now consider *N* on the space $C[0,T_{1}]$ with non-negative cone $Q=C_{+}[0,T_{1}]$. Taking $R>M$, we have shown that $u \ne {\lambda } Nu$ for all $u \in \partial Q_{R}$. By Lemma 1, *N* has a fixed point in $Q_{R}$ which is a solution of (7.1) on the interval $[0,T_{1}]$. $\square$

### Remark 7

We will have $A=a$ when $\xi \ge 0$ is a non-decreasing function, since then $I^{\alpha }\xi (t)$ is a non-decreasing function of *t*, for example see [30, Addendum].

### Corollary 1

Consider growth assumption (7.2) in the following cases.

1. $w(u)=\widehat{w}(u)=u^{r}$ for $r \le 1$. Then by Examples  1 and 2, (7.1) has a global solution, that is, exists on [0, *T*] for an arbitrary $T>0$.
2. $\widehat{w}_{k}(u)=(k^{1/p}+u)(\ln (k+u^{p}))^{1/p}$ for $k \ge 1$, or $\widehat{w}(u)= (1+u+3\sin ^2(u))(\ln (1+u^{p}))^{1/p}$; then (7.1) has a global solution by Remark 6.
3. $w(u)=\widehat{w}(u)=u^{r}$ for $r >1$; (7.1) has a local solution in this case, solution exists on some interval $[0,T_{1}]$ with $T_{1}<T$.

Another variant where we can get global solutions is by using a special case of a Gronwall inequality for singular kernel integrals due to Webb [31, Theorem 5.1].

### Theorem 5

[ [31]] Let $a>0$, $b>0$, $0<\alpha <1$ and let $\phi$ be non-increasing, $\phi \in L^1_{+}[0,T]$ for $T>0$. Suppose that $u \in C_{+}[0,T]$ satisfies the inequality

7.5 $$\begin{aligned} u(t) \le a+b \int _0^t (t-s)^{\alpha -1}\phi (s)u(s)\,ds, \; \text {for}\;t \in [0,T]. \end{aligned}$$

If $(I^{\alpha }\phi )(t) \rightarrow 0$ as $t \rightarrow 0+$, then for any $r \in (0,1)$, there exists $t_{r}>0$ such that

7.6 $$\begin{aligned} u(t)\le \frac{a}{1-r}\exp \Bigl (\frac{b}{t_{r}^{1-\alpha }(1-r)}\int _0^t \phi (s)\,ds\Bigr ), \;\text {for every}\; t>0. \end{aligned}$$

The condition $(I^{\alpha }\phi )(t) \rightarrow 0$ as $t \rightarrow 0+$ holds when $\phi \in L^{p}[0, {\tau }]$ for some $p>1/\alpha$ and some (small) ${\tau }>0$, which is similar to our general assumptions.

### Theorem 6

Suppose that $u_{0}\in \mathbb {R}_{+}$, $f \ge 0$ is $L^{p}$-Carathéodory for $p>1/\alpha$ and there exist $\xi , \phi \in L_{+}^{1}[0,T]$ with $\phi$ non-increasing and $(I^{\alpha }\phi )(t) \rightarrow 0$ as $t \rightarrow 0+$, with $I^{\alpha }\xi$ is bounded on [0, *T*] such that

7.7 $$\begin{aligned} f(t,u)\le \xi (t)+\phi (t)u\; \text {for a.e.}\; t \in [0,T]\; \text {and}\; u\in \mathbb {R}_{+}. \end{aligned}$$

Then (7.1) has a global solution *u*(*t*), that is, the solution exists on [0, *T*].

### Proof

The proof is very similar to the proof of Theorem 4, without applying Hölder’s inequality, and using the Gronwall inequality of Theorem  5 instead of the Bihari inequality to obtain an a priori bound, hence is omitted. $\square$

### Remark 8

Since $L^1$ is a larger space than $L^{p}$ for $p > 1$, the result of Theorem 6 is different from Theorem 4. When $\phi (t)= Mt^{-\gamma }$ for some $\gamma <\alpha$ and constant $M>0$, and $\xi >0$ is a constant, Theorem 6 was obtained in [29, Theorem 4.8].

### Corollary 2

Assume that $u_{0}\in \mathbb {R}_{+}$, $f \ge 0$ is $L^{p}$-Carathéodory for $p>1/\alpha$ and there exist $\rho >0$ and $\phi \in L_{+}^{p}[0,T]$ with $\int _{0}^{T}\phi ^{p}(s)\,ds>0$, such that

7.8 $$\begin{aligned} f(t,u)\le \phi (t)\widehat{w}(u)\; \text {for a.e.}\; t\in [0,T]\; \text {and}\; u\in [\rho ,\infty ), \end{aligned}$$

where $\widehat{w}:[0,\infty )\rightarrow [0,\infty )$ and $\widehat{w}^{p}(u) \le w(u^{p})$ for all $u>0$, where *w* is a non-decreasing continuous function with $w(x_0)>0$ for some $x_{0}>0$. Then (7.1) has a solution $u\in C_{+}[0,T_{1}]$, where $T_{1} \le T$ is such that the range condition as given in Theorem 4 is valid.

### Proof

Since $f \ge 0$ is $L^{p}$-Carathéodory for $p>1/\alpha$, there exists $g_{\rho }\in L_{+}^{p}[0,T]$ such that

$$\begin{aligned} |f(t,u)|\le g_{\rho }(t) \; \text {for a.e.} \;t\in [0,T] \;\text {and all}\; u\in [0, \rho ]. \end{aligned}$$

This, together with (7.8), implies

$$\begin{aligned} f(t,u) \le g_{\rho }(t) + \phi (t)\widehat{w}(u) \;\text {for a.e.} \;t\in [0,T] \;\text {and all}\; u\in \mathbb {R}_{+} \end{aligned}$$

and (7.2) holds with $\xi =g_{\rho }$. The result follows from Theorem 4. $\square$

### Corollary 3

Let $E\subset [0,T]$ be a fixed subset of measure zero. Suppose that $u_{0}\in \mathbb {R}_{+}$, $f \ge 0$ is $L^{p}$-Carathéodory for some $p>1/{\alpha }$ and there exist $r>0$, and $\psi \in L_{+}^{p}[0,T]$ with $\psi (t)>0$ for $t \in [0,T] {\setminus } E$, such that

7.9 $$\begin{aligned} \limsup _{u\rightarrow \infty }\sup _{t\in [0,T]\setminus E}\frac{f(t,u)}{u^{r}\psi (t)}<\infty . \end{aligned}$$

If $r \le 1$ then (7.1) has a global solution, the solution exist on all of [0, *T*]. If $r>1$ a local solution exists on $[0,T_{1}]$ for some $T_{1}$, as in Corollary 1.

### Proof

By (7.9), there exist $M>0$ and $\rho >0$ such that

$$\begin{aligned} f(t,u) \le M\psi (t) u^{r}\;\text {for a.e.} \; t\in [0,T] \;\text {and} \;u\in [\rho ,+\infty ). \end{aligned}$$

Hence, (7.8) with $\phi =M\psi$ and $\widehat{w}(u)= u^{r}$ holds. The result follows from Corollaries 1 and 2. $\square$

### Remark 9

We have proved results for existence of non-negative solutions. By the same methods exactly similar results hold in $C[0,T_{1}]$ by replacing *u*(*t*) by |*u*(*t*)| in the inequalities, and assuming inequalities on |*f*| such as

$$\begin{aligned} |f(t,u)|\le \xi (t)+\phi (t)\widehat{w}(|u|)\; \text {for a.e.}\; t\in [0,T]\; \text {and}\; u\in \mathbb {R}, \end{aligned}$$

in Theorem 4, similarly in Theorem 6. We do not state the obvious results.

## First order FDEs with nonlinearities from combustion theory

Motivated by some nonlinearities that arise in combustion theory, we study IVPs of first order FDEs of the form

8.1 $$\begin{aligned} D_{*}^{\alpha }u(t) =\nu \bigl (1+\varepsilon (t)u(t)\bigr )^{m}\exp \bigl ({\frac{u(t)}{1+\varepsilon (t) u(t)}}\bigr )\;\text {for a.e.} \; t \in [0,T], \end{aligned}$$

subject to the initial condition

8.2 $$\begin{aligned} u(0)=u_{0}. \end{aligned}$$

The nonlinear reaction term $f(u)=\nu (1 + \varepsilon u)^{m}\exp ({\frac{u}{1+\varepsilon u}})$ arises in combustion theory, where $\nu$ is known as the Frank-Kamenetskii parameter, *u* is the dimensionless temperature, and $\varepsilon$ the reciprocal activation energy, often taken to be a positive constant. The case $m=0$ is known as the Arrhenius reaction rate and the case $m = 1/2$ is called the bimolecular reaction rate, see [8, 28]. The reaction rate with $m<0$ is physically meaningful and has been widely studied, see [23, 27] for $m=-1$ or $m=-2$, [27] for $m\in [-2, 2.67]$, and [23] for $m\in [-10.31, 2.81]$.

The existence and uniqueness of nonzero non-negative solutions of one or higher dimensional elliptic equations in combustion theory have been widely studied, for example in [8, 9, 16, 21, 28]. For elliptic equations, $\varepsilon$ not constant corresponds to the reciprocal activation energy depending on the location.

We apply the results obtained in Sect. 7 to study a more general problem than (8.1)-(8.2), namely

8.3 $$\begin{aligned} \begin{aligned} D^{\alpha }_{*}u(t)&=\nu \bigl (1{+}\varepsilon (t)g(u(t)\bigr )^{m}\exp \Bigl ({\frac{h(u(t))}{1{+}\varepsilon (t)h(u(t))}}\Bigr ){:=}f(t,u(t)), \;\text {a.e.} \; t\in [0,T],\\&\;\;u(0)=u_{0}, \end{aligned}\nonumber \\ \end{aligned}$$

where $u_{0} \ge 0$, $\nu >0$, $m > 0$, $\varepsilon \in L_{+}^{p/m}$ for some $p>1/\alpha$, with $\varepsilon (t) \ge \varepsilon _{0}>0$, *g* is continuous, $g(u) \ge 0$ and is non-decreasing for $u \ge 0$, and *h* is continuous with $h(u) \ge 0$ for $u \ge 0$.

We say a solution $u \in C_{+}[0,T_{1}]$ is positive if $u(t)>0$ for $t \in (0,T_{1}]$.

### Theorem 7

Under the above hypotheses the IVP (8.3) has a positive solution defined on some interval $[0,T_{1}]$.

### Proof

Note that *f* satisfies the Carathéodory conditions since *g*, *h* are continuous. Also we have

$$\begin{aligned} \exp \bigl ({\frac{h(u(t))}{1+\varepsilon (t) h(u(t))}}\bigr ) \le \exp \bigl (\frac{1}{\varepsilon (t)}\bigr ) \le \exp \bigl (\frac{1}{\varepsilon _{0}}\bigr ), \;\;\text {for all}\;t. \end{aligned}$$

For $M_{1}:=\max \{1,2^{m-1}\}$, we have

$$\begin{aligned} f(t,u) \le \nu \exp (1/{\varepsilon _{0}})(1+{\varepsilon (t)}g(u(t))^{m} \le \nu \exp (1/{\varepsilon _{0}})M_{1}\bigl (1+{\varepsilon ^{m}(t)}g^{m}(u(t))\bigr ), \end{aligned}$$

hence *f* is $L^{p}$-Carathéodory since $\varepsilon ^{m} \in L^{p}$. Define

8.4 $$\begin{aligned} \xi = \nu \exp (1/{\varepsilon _{0}})M_{1}, \;\phi (t):={\nu }\exp (1/{\varepsilon _{0}})M_{1}\varepsilon ^{m}(t),\; w(u)= \widehat{w}(u):=g^{m}(u).\nonumber \\ \end{aligned}$$

Thus $f(t,u) \le \xi + {\phi (t)} w(u)$ with $\phi \in L^{p}$, *w* continuous and non-decreasing. By Theorem 4 the problem has a solution which exists on some interval $[0,T_{1}]$. Since $Fu(t)>0$ for every $t \in [0,T_{1}]$ and $u=u_{0}+I^{\alpha }Fu$, the solution satisfies $u(t)> u_{0}$ for every $t \in (0,T_{1}]$. $\square$

### Remark 10

For a fixed $\alpha \in (0,1)$ it cannot be inferred from $D^{\alpha }_{*}u(t)>0$ that the solution *u* is increasing, as shown by counter examples in [5, Example 2.1] and [30, Proposition 7.2]. Monotonicity of functions related to the signs of their Caputo derivatives is discussed in detail in [5].

We give a general example to illustrate how we can get global solutions in some cases when $m \in \mathbb {R}$ and the reciprocal activation energy $\varepsilon$ is a function of *t*, that is, we consider the problem

8.5 $$\begin{aligned} D_{*}^{\alpha }u(t) {=}\nu (1+\varepsilon (t) u^{r}(t))^{m}\exp \Bigl ({\frac{h(u(t))}{1{+}\varepsilon (t)h(u(t))}}\Bigr )\;\text {for a.e.} \; t \in [0,T],\;\; u(0){=}u_{0},\nonumber \\ \end{aligned}$$

where $\nu ,r$ are positive parameters, $m \in \mathbb {R}$, *h* is continuous and $h(u) \ge 0$ for $u \ge 0$, and $\varepsilon (t)\ge \varepsilon _{0}$ for some $\varepsilon _{0}>0$, also $\varepsilon ^{m}\in L^{p}[0,T]$ when $m >0$ for some $p>1/\alpha$.

### Theorem 8

Assume that above hypotheses hold and $u_{0}\ge 0$.

(1) If $rm\in (-\infty , 1]$, then (8.5) has a global positive solution, that is, for arbitrary $T>0$, a positive solution exists on [0, *T*].

(2) If $rm>1$ then (8.5) has a positive solution on some interval $[0,T_{1}]$

### Proof

Let $f:[0,T] \times \mathbb R_{+}\rightarrow \mathbb R_{+}$ be defined by

8.6 $$\begin{aligned} f(t,u)=\nu (1+\varepsilon (t) u^{r})^{m}\exp \Bigl ({\frac{h(u(t))}{1+\varepsilon (t)h(u(t))}}\Bigr ). \end{aligned}$$

If $m \le 0$ then $(1+\varepsilon (t)u^{r})^{m} \le 1$ and $f(t,u) \le \nu \exp (1/{\varepsilon _{0}})$, so *f* is globally bounded and a solution exists on [0, *T*]. Now suppose that $m>0$. Let $M_{1}:=\max \{1,2^{m-1}\}$, then we have

8.7 $$\begin{aligned} f(t,u) \le \nu \exp (1/{\varepsilon _{0}}) (1+\varepsilon (t) u^{r})^{m} \le \nu \exp (1/{\varepsilon _{0}})M_{1}(1+\varepsilon ^{m}(t)u^{rm}), \end{aligned}$$

hence *f* is $L^{p}$-Carathéodory. Let $\xi ={\nu }\exp (1/{\varepsilon _{0}})M_{1}$, $\phi (t)={\nu } \exp (1/{\varepsilon _{0}})M_{1} \varepsilon ^{m}(t)$ and $w(u)=u^{rm}$. From (8.7) we see that, for case (1), $mr \le 1$, Corollary 1 1 applies, a solution exists on [0, *T*], and is positive for $t>0$. For case (2), $mr>1$, Corollary 1 3 applies and we conclude that there is a solution in $C_{+}[0,T_{1}]$ for some interval $[0,T_{1}]$. $\square$

### Remark 11

Clearly we could replace $u^{r}$ in this example by a function *g*(*u*) under suitable restrictions.

## The Caputo fractional derivative

The Caputo fractional derivative is frequently defined with the derivative and fractional integral taken in the reverse order to that of the R-L derivative, that is as $\widehat{D}_{C}^{\alpha }u(t):=I^{1-\alpha }u'(t)$. For this fractional integral to be defined we require $u' \in L^1$. Therefore we have

**Provisional Definition** For $\alpha \in (0,1)$ and $u' \in L^1[0,T]$ the Caputo fractional derivative $\widehat{D}_{C}^{\alpha }u$ is defined for a.e. *t* by

$$\begin{aligned} \widehat{D}_{C}^{\alpha }u(t):=(I^{1-\alpha }u')(t). \end{aligned}$$

A severe problem is that there exist singular functions where the derivative exists a.e., for example Lebesgue’s singular function $\varphi$ (also known as the Cantor-Vitali function, or Devil’s staircase) which is (Hölder) continuous but not *AC*, $\varphi (0)=0, \varphi (1)=1$, and $\varphi '(t)=0$ for a.e. *t*, so we would have $\widehat{D}_{C}^{\alpha }\varphi (t)=0, \varphi (0)=0$. Hence this operator can never be invertible, the problem $\widehat{D}_{C}^{\alpha }u(t)=0, u(0)=0$ has infinitely many non-zero solutions.

It has often been claimed that for $0<\alpha <1$ and *f* continuous

9.1 $$\begin{aligned} \widehat{D}_{C}^{\alpha }u(t)=f(t), \; u(0)=u_{0} \;\text {is equivalent to} \;u(t)=u_{0}+I^{\alpha }f. \end{aligned}$$

However, the ‘solution’ on each side of the equation (9.1) has different meanings. Usually a solution of the equation $u(t)=u_{0}+I^{\alpha }f$ is a function in *C*[0, *T*]. If $f \in L^1$ and $u \in C[0,T]$ satisfies $\widehat{D}_{C}^{\alpha }u(t)=f(t)$ a.e., then, by the definition, $I^{1-\alpha }u'(t)=f(t)$ a.e., and clearly $f \in I^{1-\alpha }(L^1)$. By the semigroup property, $I^{\alpha }f(t)=(Iu')(t)$ so that $I^{\alpha }f \in AC$. Thus to have any hope of an equivalence being valid we must have $u=u_{0}+I^{\alpha }f \in AC$.

Therefore the definition must be as follows.

### Definition 8

For $\alpha \in (0,1)$ and $u \in AC[0,T]$ the Caputo fractional derivative $D_{C}^{\alpha }u$ is defined by

$$\begin{aligned} D_{C}^{\alpha }u(t):=I^{1-\alpha }u'(t), \;\;\text {a.e.} \;t \in [0,T]. \end{aligned}$$

For $u \in AC$, $u' \in L^1$ and so $D_{C}^{\alpha }u=I^{1-\alpha }(u')$ is defined as an $L^1$ function, hence $D_{C}^{\alpha }u(t)$ is defined for a.e. *t*.

This definition prevents having functions such as Lebesgue’s singular function. Moreover, it now follows simply that if $u \in AC$, $f \in C[0,T]$ and $D^{\alpha }_{C}u(t)=f(t)$ a.e. and $u(0)=u_0$ then indeed $u(t)=u_0+I^{\alpha }f(t)$, as in the first part of the proof of Theorem 9 below.

However, the converse fails. If $u \in C[0,T]$ is a solution of $u(t)=u_{0}+I^{\alpha }f(t)$ with *f* continuous, then clearly $u \in AC$ if and only if $I^{\alpha }f \in AC$. This has been claimed many times but has never been proved for a very good reason: it is false in general, so the derivative $D^{\alpha }_{C}u(t)$ is not shown to exist. $I^{\alpha }$ does not map all of *C*[0, *T*] into *AC*[0, *T*] as shown in Cichoń-Salem [4, Counter-Example 1], and independently in [30, Addendum] who quoted an example of a Weierstrass type function from Hardy and Littlewood [13, §5.5]. Thus *f* continuous and $u(t)=u_{0}+I^{\alpha }f(t)$ does not imply that $u \in AC$ without extra conditions on *f*. In other words, it is impossible to prove the often claimed equivalence (9.1).

To get a useful equivalence it is necessary to use the definition $D_{*}^{\alpha }u$ of Caputo differential operator as done in Diethelm’s book [6] and as we have done. As shown in Diethelm [6, Theorem 3.1], or [30, Proposition 4.4], for $0<\alpha <1$ the two definitions $D^{\alpha }_{C}u$ and $D_{*}^{\alpha }u$ coincide when $u \in AC$, so there is no reason to consider $D^{\alpha }_{C}u$ as defined in Definition 8.

We do have an equivalence result, with a necessary condition $f\in I^{1-\alpha }(L^1)$, but it not known how it can be applied in the important case when *f*(*t*) is replaced by *f*(*t*, *u*(*t*)) when *u* is only known to be continuous, see Remark 13 below. The result can be proved using [18, Theorem 4.4] and [18, Corollary 4.3]. We give a direct proof to emphasize the necessary condition.

### Theorem 9

Let $u \in AC$, $f \in L^1$ and $D^{\alpha }_{C}u(t)=f(t)$ for a.e. *t* and $u(0)=u_0$. Then $f\in I^{1-\alpha }(L^1)$ and $u(t)=u_0+I^{\alpha }f(t)$ for all $t\in [0,T]$. Conversely, if $f\in I^{1-\alpha }(L^1)$ and *u* is continuous and satisfies $u(t)=u_0+I^{\alpha }f(t)$ for $t \in [0,T]$, then $u \in AC$, $D^{\alpha }_{C}u(t)=f(t)$ for a.e. *t*, and $u(0)=u_0$.

### Proof

If $u \in AC$ and $D^{\alpha }_{C}u(t)=f(t)$ for a.e. *t*, then $I^{1-\alpha }u'=f$ thus $f \in I^{1-\alpha }(L^1)$, say $f=I^{1-\alpha }g$ for $g \in L^1$ (of course $g=u'$ here). Applying the operator $I^{\alpha }$ and using the semigroup property gives $Iu'=I^{\alpha }f=Ig$. Since $u \in AC$, $Iu'(t)=u(t)-u(0)=Ig=I^{\alpha }f$. Conversely, if $f\in I^{1-\alpha }(L^1)$, say $f=I^{1-\alpha }g$ for $g \in L^1$, and *u* is continuous and satisfies $u(t)=u_0+I^{\alpha }f(t)$, then $u=u_0+I^{\alpha }f=u_0+Ig \in AC$ and $u'=g$ a.e.. Thus $u(0)=u_0$ and $D^{\alpha }_{C}u=I^{1-\alpha }u'=I^{1-\alpha }g=f$ a.e.. $\square$

In particular this gives a nonexistence result.

### Proposition 3

If $f \in C\setminus I^{1-\alpha }(L^1)$, then the IVP: $D^{\alpha }_{C}u(t)=f(t)$ for a.e. *t*, $u(0)=u_0$, has no solutions.

As mentioned above, there exist continuous functions *f* such that $I^{\alpha }f \notin AC$, therefore $C\setminus I^{1-\alpha }(L^1)$ is nonempty.

### Remark 12

A paper of Vainikko [26] investigates the existence of fractional derivatives particularly when such derivatives are continuous. For example, Theorem 5.2 of that paper shows that having a function $v \in I^{\alpha }(C[0,T])$, that is $v=I^{\alpha }g$ for some $g \in C[0,T]$, is equivalent to some structure conditions and also to having $D^{\alpha }_{*}v$ continuous. In this case $v=I^{\alpha }g$ is Hölder continuous, so $v(0)=0$ and then $D^{\alpha }_{*}v(t)=D(I^{1-\alpha }v)(t)=D(Ig)(t)=g(t)$ for every *t* since $Ig \in C^1$, which shows that $D^{\alpha }_{*}v$ is continuous. However we do not know that $v'$ is in AC so $D^{\alpha }_{C}v$ might not exist. When $u \in C[0,T]$ is a solution of the Volterra integral equation $u(t)=u_{0}+I^{\alpha }Fu$, where *F* is the Nemytskii operator, and *f* is continuous, *Fu* is continuous and this would give $D^{\alpha }_{*}u$ is continuous. For the case we study, *f* is $L^{p}$-Carathéodory, we only have $Fu \in L^{p}$ and $D^{\alpha }_{*}u \in L^1$.

### Remark 13

When we consider *f*(*t*, *u*(*t*)) with *u* continuous, *f*(*t*) is replaced by *Fu*(*t*). Then a necessary and sufficient condition for $D^{\alpha }_{C}u(t)=Fu(t)$, $u(0)=u_0$, to have solutions is that $Fu \in I^{1-\alpha }(L^1)$; Proposition 2 shows that this requires $I^{\alpha }Fu \in AC$. This is a strong condition, and when *u* is only known to be continuous, explicit assumptions on *f* to achieve this are not known, for example $f\in C^{\infty }$ does not imply this. There are many papers in the literature, too many to be cited in this paper, where solutions of $D^{\alpha }_{C}u(t)=f(t,u(t)), \; u(0)=u_{0}$ (or $\widehat{D}^{\alpha }_{C}u(t)=f(t,u(t))$, or even less precise definitions) are studied via solutions *u* in *C*[0, *T*] of the integral equation $u(t)=u_{0}+I^{\alpha }Fu(t)$, when *f* is only assumed to be continuous, but these papers do not consider the necessary condition $Fu \in I^{1-\alpha }(L^1)$. In fact this ‘false equivalence’ is often used as a first step in studying many types of problems involving the Caputo fractional derivative, such as boundary value problems. Therefore, the claimed results in these paper are based on a false premise.
